# Effect of Rice *GDP-L-Galactose Phosphorylase* Constitutive Overexpression on Ascorbate Concentration, Stress Tolerance, and Iron Bioavailability in Rice

**DOI:** 10.3389/fpls.2020.595439

**Published:** 2020-12-03

**Authors:** Ronan C. Broad, Julien P. Bonneau, Jesse T. Beasley, Sally Roden, Pawel Sadowski, Nathaniel Jewell, Chris Brien, Bettina Berger, Elad Tako, Raymond P. Glahn, Roger P. Hellens, Alexander A. T. Johnson

**Affiliations:** ^1^School of Biosciences, The University of Melbourne, Melbourne, VIC, Australia; ^2^Centre for Agriculture and the Bioeconomy, Institute for Future Environments, Queensland University of Technology, Brisbane, QLD, Australia; ^3^Central Analytical Research Facility, Institute for Future Environments, Queensland University of Technology, Brisbane, QLD, Australia; ^4^Australian Plant Phenomics Facility and School for Agriculture, Food and Wine, The University of Adelaide, Adelaide, SA, Australia; ^5^Department of Food Science, Cornell University, Ithaca, NY, United States; ^6^Robert W. Holley Center for Agriculture and Health, USDA-ARS, Ithaca, NY, United States

**Keywords:** ascorbic acid, vitamin C, metabolic engineering, genetic engineering, biofortification, abiotic stress, nutrition

## Abstract

Ascorbate (vitamin C) is an essential multifunctional molecule for both plants and mammals. In plants, ascorbate is the most abundant water-soluble antioxidant that supports stress tolerance. In humans, ascorbate is an essential micronutrient and promotes iron (Fe) absorption in the gut. Engineering crops with increased ascorbate levels have the potential to improve both crop stress tolerance and human health. Here, rice (*Oryza sativa* L.) plants were engineered to constitutively overexpress the rice *GDP-L-galactose phosphorylase* coding sequence (35S-*OsGGP*), which encodes the rate-limiting enzymatic step of the L-galactose pathway. Ascorbate concentrations were negligible in both null segregant (NS) and 35S-*OsGGP* brown rice (BR, unpolished grain), but significantly increased in 35S-*OsGGP* germinated brown rice (GBR) relative to NS. Foliar ascorbate concentrations were significantly increased in 35S-*OsGGP* plants in the vegetative growth phase relative to NS, but significantly reduced at the reproductive growth phase and were associated with reduced *OsGGP* transcript levels. The 35S-*OsGGP* plants did not display altered salt tolerance at the vegetative growth phase despite having elevated ascorbate concentrations. Ascorbate concentrations were positively correlated with ferritin concentrations in Caco-2 cells – an accurate predictor of Fe bioavailability in human digestion – exposed to *in vitro* digests of NS and 35S-*OsGGP* BR and GBR samples.

## Introduction

Producing sufficient and nutritious food to feed the growing world population represents one of the great challenges of the 21st century. Abiotic stresses, such as drought, salinity, and extreme temperatures are major limiting factors of global crop productivity, and are predicted to be exacerbated and co-occur more frequently due to the effects of climate change (Zhang et al., 2000; Pandey et al., 2017). Common to many abiotic stresses is the excess production of reactive oxygen species (ROS) due to the disruption of cellular homeostasis (Mittler, 2002). The development of crops with an enhanced capacity to mitigate the damaging effects caused by the excess production of ROS represents one solution to help offset the forecasted yield losses associated with climate change (Koyro et al., 2012). Micronutrient deficiencies – a form of undernutrition commonly referred to as hidden hunger – are a major threat to the health and development of populations worldwide, affecting more than 2 billion people (von Grebmer et al., 2014). Hidden hunger is most prominent in the developing world, often resulting from diets dependent micronutrient-poor staple crops (von Grebmer et al., 2014). The production of crops containing increased micronutrient density and/or bioavailability – a process known as biofortification – offers a cost-effective and sustainable intervention to reduce hidden hunger (Bouis et al., 2011).

Ascorbate (also known as vitamin C) is a vital multifunctional molecule for both plants and mammals (Smirnoff, 2018). Ascorbate is a reducing agent capable of donating electrons, and primarily serves a role as a cellular antioxidant and enzymatic co-factor (Smirnoff, 2018). In plants, ascorbate performs crucial roles in photosynthetic functions and stress tolerance, largely due to its ability to combat excess ROS produced by normal or stressed cellular metabolism as a ROS scavenger or through the ascorbate-glutathione cycle – an indispensable antioxidant system for controlling cellular redox homeostasis (Foyer and Noctor, 2011). Beyond its functions in photosynthesis and stress tolerance, ascorbate plays many other roles in plants, including the biosynthesis of ethylene and organic acids (Rocklin et al., 1999; Debolt et al., 2006), regenerating oxidized *α*-tocopherol (Fryer, 1992), transporting iron (Fe; Grillet et al., 2014), and influencing cell division (Potters et al., 2002), cell expansion (Smirnoff, 1996), and the onset of flowering and senescence (Barth et al., 2006). In humans, ascorbate is an essential micronutrient well-known for its essential role in collagen biosynthesis and prevention of the disease scurvy (Peterkofsky, 1991). Ascorbate also plays many other key roles in human physiology, including the cellular uptake of dietary Fe in the gut. Ascorbate is a powerful enhancer of Fe bioavailability – the proportion of Fe that is absorbed and is available for use or storage – and can overcome Fe absorption inhibitors such as phytate and polyphenolic compounds (Fairweather-Tait, 1987; Hallberg et al., 1989; Siegenberg et al., 1991; Engle-Stone et al., 2005; Jin et al., 2008; Kim et al., 2011). In addition to enhancing Fe absorption in the gut, ascorbate has also been reported to regulate cellular Fe uptake and metabolism (Lane et al., 2013; Lane and Richardson, 2014). Ascorbate has many further positive effects on human health, for instance by reducing the risk of infections, cardiovascular disease, stroke, and cancer (Combs, 2012).

Several routes toward ascorbate biosynthesis have been proposed in plants, however, substantial genetic evidence supports the L-galactose pathway – which converts D-fructose-6-P to ascorbate *via* eight enzymatic steps – as the predominant pathway toward ascorbate biosynthesis in plants (Dowdle et al., 2007; Baldet et al., 2013; Höller et al., 2015; Lim et al., 2016a; Vidal-Meireles et al., 2017). The *GPD-L-galactose phosphorylase* (*GGP*, also known as *VTC2*/*VTC5*) gene encodes the fifth enzymatic step of the L-galactose pathway catalyzing the conversion of GDP-L-galactose to L-galactose1-P and represents the first committed step toward ascorbate biosynthesis. Relative to other genes from the L-galactose pathway, increased expression of the *GGP* gene consistently results in the largest increases in ascorbate concentrations in a wide range of species, providing strong genetic evidence for GGP as the rate-limiting enzymatic step of the L-galactose pathway. For example, increased expression of the *GGP* gene has increased ascorbate concentrations 2.9- to 4.1-fold in *Arabidopsis thaliana* (Bulley et al., 2009; Zhou et al., 2012), 3.1-fold in potato (*Solanum tuberosum* L. Bulley et al., 2012), 2.1-fold in strawberry (*Fragaria* × *ananassa*; Bulley et al., 2012), 2.0- to 6.2-fold in tomato (*Solanum lycopersicum* L.; Bulley et al., 2012; Li et al., 2019), 1.4-fold in tobacco (*Nicotiana tabacum* L.; Wang et al., 2014), and 2.5- to 2.6-fold in rice (*Oryza sativa* L.; Zhang et al., 2015; Ali et al., 2019). Increased expression of the *GGP* gene has further been associated with enhanced tolerance to multiple abiotic stresses, including salt, ozone, and cold stress (Wang et al., 2014; Zhang et al., 2015; Ali et al., 2019). Although many studies have increased ascorbate concentrations in model and crop species, none have investigated whether the elevated levels of ascorbate might improve Fe bioavailability in human digestion.

Rice is the most important source of calories for humans, providing nearly half the world’s population with more than 20% of their daily caloric intake (Bonneau et al., 2018). It is an important staple crop for many parts of the world, particularly in Eastern Asia, Southern Asia, and South-East Asia (Bonneau et al., 2018). Abiotic stresses, such as drought, flooding, and salinity are major limiting factors in global rice productivity (Lafitte et al., 2004), therefore developing strategies to increase ascorbate concentrations in rice plants may help to mitigate the yield losses associated with these abiotic stresses. Moreover, rice grain contains negligible concentrations of ascorbate and is a poor source of bioavailable Fe (Haas et al., 2005; USDA, 2019), thus strategies to increase ascorbate levels in rice grain could improve dietary intakes of both ascorbate and bioavailable Fe in humans. To date, several studies have increased the expression of ascorbate biosynthetic or recycling genes to increase foliar ascorbate concentrations in rice plants (Liu et al., 2011; Kim et al., 2013; Zhang et al., 2015; Ali et al., 2019). Whether these strategies alter ascorbate concentrations in the rice grain and/or other tissues are unclear.

In this study, we aimed to increase ascorbate concentrations throughout the rice plant *via* constitutive overexpression of the *OsGGP* coding sequence and to determine the effect that elevated levels of ascorbate have on both stress tolerance and Fe bioavailability. We found that constitutive overexpression of the *OsGGP* coding sequence in rice did not affect ascorbate concentrations in brown rice (BR, unpolished grain), but significantly increased ascorbate concentrations in germinated brown rice (GBR) and tissues at the vegetative growth phase. At the reproductive growth phase, ascorbate concentrations were significantly reduced in homozygous 35S-*OsGGP* plants and were associated with significantly reduced transcript levels of the endogenous *OsGGP* gene. Automated imaging revealed that the 35S-*OsGGP* plants did not display increased salt tolerance at the vegetative growth phase, despite having elevated ascorbate concentrations. Finally, we found that ascorbate concentrations were positively correlated with ferritin concentrations in Caco-2 cells – an accurate predictor of Fe bioavailability in human digestion – exposed to *in vitro* digests of null segregant (NS) and 35S-*OsGGP* BR and GBR samples.

## Materials and Methods

### Plant Growth Conditions

*Oryza sativa* cv. Nipponbare was used for all experiments. Rice grain was surface sterilized with 70% (v/v) ethanol (Chem-Supply, SA, Australia) for 1 min and 30% (v/v) bleach (White King, NSW, Australia) with a few drops of Tween-20 (Sigma-Aldrich, MO, United States) for 30 min and washed three times with sterile dH_2_O. Surface sterilized rice grain was germinated in a petri dish with moist sterile filter paper (Whatman, United Kingdom) for 7–9 days prior to transplanting to 1 l pots filled with potting mix in a glasshouse maintained at 26°C and 70% relative humidity at The University of Melbourne (Melbourne, VIC, Australia). Lighting was provided through natural lighting supplemented with a mixture of high-pressure sodium and metal halide lamps for 12 h during the day. The potting mix was prepared by mixing one part washed fine sand (Col Smith, VIC, Australia), one part propagating sand (Brunnings, VIC, Australia), two parts premium vermiculate (Exfoliators, VIC, Australia), and one part General Mix potting media (Australian Growing Solutions, VIC, Australia) fertilized with Osmocote Exact Standard 8–9 M (ICL, NSW, Australia) at a rate of 6 g/L.

### Vector Construction and Rice Transformation

The *OsGGP* (LOC_Os12g08810) coding sequence was PCR-amplified from rice cv. Nipponbare cDNA and recombined into the Gateway-compatible pMDC32 vector (Curtis and Grossniklaus, 2003), which placed the *OsGGP* coding sequence under transcriptional control of the constitutive dual CaMV 35S promoter (Battraw and Hall, 1990; Terada and Shimamoto, 1990) and adjacent to the *hygromycin phosphotransferase II* (*hptII*) plant-selectable marker gene (Supplementary Table S1). *Agrobacterium*-mediated transformation of rice callus was carried out using established protocols (Sallaud et al., 2003).

### Genotyping

Genomic DNA was extracted using the Extract-N-Amp™ Plant PCR Kit (Sigma-Aldrich) according to manufacturer’s instructions. Presence of the 35S-*OsGGP* transfer DNA (T-DNA) was determined *via* multiplex PCR amplification of *hptII* with *OsACT1*. The PCR amplification cycles consisted of 1 cycle = 1 min 95°C; 40 cycles = 20 s 95°C, 20 s 60°C, and 90 s 72°C. The PCRs were performed in a final volume of 20 μl for MyTaq™ HS Red DNA Polymerase (Bioline, United Kingdom) according to manufacturer’s instructions.

### Quantification of Total Ascorbate

Total ascorbate was extracted and measured as previously described with modifications (Rassam and Laing, 2005). Briefly, total ascorbate was extracted from ground, lyophilized tissue homogenized in extraction fluid containing 8% metaphosphoric acid, 2 mM EDTA, and 2 mM TCEP (Sigma-Aldrich) at 40°C for 2 h. The extract was centrifuged and 5 μl of supernatant was injected onto a C18 3 μm 33 × 7 mm Alltima Rocket column (Hichrom Limited, United Kingdom) maintained at 40°C with a flow rate of 1 ml/min. The concentration was determined by reverse phase chromatography on a Shimadzu Nexera UHPLC system (Shimadzu, Japan) with simultaneous UV and MS detection. Mobile phase A consisted of MS grade water with 0.1% formic acid (ThermoFisher Scientific, MA, United States). The elution procedure utilized a 1 min gradient from 0 to 90% of mobile phase B: MS grade acetonitrile with 0.1% formic acid (ThermoFisher Scientific). The UV absorption signal was acquired using a Shimadzu SPD-20A detector at 245 nm wavelength. The MS data were acquired on a Shimadzu LCMS-8050 triple quadrupole mass spectrometer equipped with a DUIS source. The MS instrument was operated in Multiple Reaction Monitoring mode monitoring ions in both positive (177 > 95, 177 > 85) and negative (175 > 115, 175 > 71, and 175 > 59) ionization modes. The product ion 115 m/z was used as a quantifier. A calibration curve in the range of 1.95–250 ppm of L-ascorbic acid analytical standard (Sigma-Aldrich) was used to determine absolute concentration of analyte in the extracts.

### Quantitative Reverse Transcription-PCR Analysis

Total RNA from foliar tissue was isolated using the Direct-zol™ RNA MiniPrep Kit (Zymo Research, CA, United States) according to manufacturer’s instructions. To isolate total RNA from BR and GBR, sub-samples of snap-frozen BR and GBR were ground using a Tube Mill with a chilled 40 ml grinding chamber (IKA, Germany) and suspended in an extraction buffer containing 0.1 M glycine-NaOH (Chem-Supply), 100 mM NaCl (Chem-Supply), 10 mM EDTA (Chem-Supply), 2% SDS (Sigma-Aldrich), and 1% sodium lauryl-sarcosine (Sigma-Aldrich) at pH 9 for 7 min at 1,400 RPM (Nagy et al., 2016). The solution was then mixed with an equal volume of phenol to chloroform to isoamyl alcohol at a ratio of 25:24:1 (Sigma-Aldrich) for 1 min at 1,400 RPM followed by incubation on ice for 5 min. After centrifugation for 20 min at 4°C, total RNA was then purified from the upper aqueous phase using the Direct-zol™ RNA MiniPrep Kit (Zymo Research) according to manufacturer’s instructions. Quantification, DNase treatment, and reverse transcription of the RNA were performed using the QuantiFluor® RNA System (Promega, WI, United States), RQ1 RNase-Free DNase (Promega), and the Tetro cDNA Synthesis Kit (Bioline), respectively, according to manufacturer’s instructions. The quantitative reverse transcription-PCR (qRT-PCR) primers were designed to amplify the *OsGGP*, 35S-*OsGGP*, *OsGME1*, *OsGME2*, and *OsGPP* genes using Primer 3 software (Koressaar and Remm, 2007; Untergasser et al., 2012; Supplementary Table S1). To discriminate between the *OsGGP* and 35S-*OsGGP* transcripts, the qRT-PCR-*OsGGP* reverse primer was designed in the 3' untranslated region of the endogenous *OsGGP* transcript, and the qRT-PCR-35S-*OsGGP* reverse primer was designed in the nopaline synthase terminator of the transgenic 35S-*OsGGP* transcript. Primer efficiency was ≥93% for all primer pairs. The qRT-PCR analysis was carried out on a CFX Connect™ Real-Time System (Bio-Rad, CA, United States) with Hard-Shell® 96-well PCR plates (Bio-Rad). The qRT-PCR amplification cycles consisted of 1 cycle = 3 min 95°C; 40 cycles = 20 s 95°C, 20 s 60°C, and 20 s 72°C and were followed by a melt curve from 65 to 95°C in 0.5°C steps for 5 s/step. The qRT-PCRs were performed in a final volume of 10 μl for KAPA SYBR® FAST (Kapa Biosystems, MA, United States) according to manufacturer’s instructions, each with four technical replicates. The absolute quantification of transcript copy number was determined using a 10-fold serial dilution of 10^8^–10^2^ copies for each PCR product and a standard curve generated with the Bio-Rad CFX Manager 3.1 software (Bio-Rad). The PCR products were purified and quantified with a DNA Clean & Concentrator™-5 Kit (Zymo Research) and the QuantiFluor® ONE dsDNA System (Promega), respectively, prior to serial dilution. The geometric mean expression of three optimal housekeeping genes selected from *OsGAPDH*, *OsELF1*, *OsACT1*, and *OsUBQ5* using the NormFinder Excel add-in (Andersen et al., 2004) was used to normalize the expression of the 35S-*OsGGP*, *OsGGP*, *OsGME1*, *OsGME2*, and *OsGPP* genes.

### Assessment of Salt Tolerance With Automated Imaging

Surface sterilized rice grain was germinated in plastic boxes on moist paper towels [January 9, 2019, denoted days after planning (DAP) 0]. At DAP 6, three uniformly germinated grain per line were transplanted to 2.8 L pots filled with 2.6 kg of University of California (UC) Davis Soil Mix and placed into square containers so that any water from the automated watering system that drains from the bottom of the pots would be collected. The UC Davis Soil Mix was supplied by the South Australian Research and Development Institute (SARDI), Waite Campus and was prepared by mixing sand and peat moss (volume ratio 1.6:1) fertilized with Mini Osmocote (ICL) at a rate of 2.5 g/L and the pH adjusted to 6–6.5 with a mix of calcium hydroxide and calcium carbonate. Plants were grown on benches at the back of the North West automated greenhouse (NW Smarthouse) at The Plant Accelerator, University of Adelaide, Australia under manual watering (not to weight). The temperature throughout the experiment was at 28/23°C day/night with an average relative humidity of 60%. Lighting was provided through natural lighting (no supplemental lighting) with an average maximum light intensity at solar noon of 650 μmol m^−2^ s^−1^ and 14.25 h day length at the beginning of the experiment and 13.25 h day length at completion. At DAP 14, plants were thinned to one seedling per pot and loaded onto the conveyor system (LemnaTec GmbH, Aachen, Germany) of the NW Smarthouse for automated imaging. A split-unit design was used to randomize pots (i.e., line and treatment combinations) to cart positions within the Smarthouse. The plants were automatically watered daily so that 500 ml of water was maintained in the soil. The salt plants were treated with saline solution on DAP 20 and 23 (100 ml of 225 mM NaCl) to reach a final concentration of 90 mM NaCl in the soil solution, while the control plants received equivalent water applications as previously described (Campbell et al., 2015). Imaging was carried out daily from DAP 14 to DAP 40 inclusive. From these images, the projected shoot area (PSA) of the plant shoot, as viewed using RGB cameras, was obtained as previously described (Pham et al., 2019). For analysis purposes, PSA was defined as the sum of the areas as measured (in kilopixels) from six side/oblique camera views plus twice the shoot area of the top view. The data were prepared for analysis using growthPheno (Brien, 2019b), a package for the R statistical computing environment (R Core Team, 2019).

### Preparation of Germinated Brown Rice

Following surface sterilization, BR samples were immediately snap-frozen in liquid nitrogen, lyophilized, and ground using a Tube Mill with a 40 ml grinding chamber (IKA), whereas GBR samples were imbibed in sterile dH_2_O for 12 h in the dark at 28°C, washed three times with sterile dH_2_O, and spread in a petri dish with moist sterile filter paper (Whatman) for 72 h in the dark at 28°C. The GBR was then washed three times with sterile dH_2_O, snap-frozen in liquid nitrogen, lyophilized, and ground using a Tube Mill with a 40 ml grinding chamber (IKA).

### Quantification of Fe

Inductively coupled plasma optical emission spectrometry (ICP-OES) of the ground, lyophilized BR and GBR samples were conducted at the Robert W. Holley Centre for Agriculture and Health (USDA-ARS, Ithaca, NY, United States) to determine Fe concentrations as previously described (Dias et al., 2018).

### Quantification of Phytate

Phytate concentrations of the ground, lyophilized BR and GBR samples were measured as the proportion of phosphorus released by phytase and alkaline phosphatase using the Phytic Acid/Total Phosphorus Assay Kit (Megazyme International, Ireland). Phosphorus concentrations in free and total phosphorus solutions were determined by absorbance at 655 nm.

### Caco-2 Cell Culture Assessment of Fe Bioavailability

Ground, lyophilized BR and GBR samples underwent an *in vitro* gastrointestinal digestion using porcine pepsin, pancreatin, and bile extract (Sigma-Aldrich) prior to Caco-2 cell Fe bioavailability analysis as previously described (Glahn et al., 1998; Trijatmiko et al., 2016; Beasley et al., 2019). Briefly, the Caco-2 cells were maintained at 37°C in supplemented Dulbecco’s modified Eagle medium (ThermoFisher Scientific) for 11 days post-seeding and replaced with supplemented minimum essential media (MEM) solution (ThermoFisher Scientific) 48 h prior to the experiment day. On the experiment day, the *in vitro* gastrointestinal-digested samples were added to cylindrical Transwell inserts (Corning Life Sciences, NY, United States) fitted with a Spectra/Por 2.1 15,000 Da molecular weight cut-off dialysis membrane (Spectrum Medical, CA, United States). The inserts were placed within wells containing Caco-2 cell monolayers and incubated for 2 h at 37°C, after which the inserts were removed, and additional MEM was added to the cells before incubation for 22 h at 37°C. After incubation, the growth medium was removed by aspiration, and the Caco-2 cells were washed twice with a rinse solution containing 140 mM/L NaCl, 5 mM/L KCl, and 10 mM/L PIPES (Sigma-Aldrich) at pH 7 and harvested with the addition of dH_2_O followed by brief sonication. From an aliquot of the Caco-2 cell solution, ferritin and protein content was determined using a FER-IRON II Ferritin Assay (Ramco Laboratories, TX, United States) and *DC*™ Protein Assay (Bio-Rad), respectively, according to manufacturer’s instructions. As Caco-2 cells synthesize ferritin in response to intracellular Fe, the ratio of ferritin/total protein (expressed as ng ferritin/mg protein) was used as an index of cellular Fe uptake (Glahn et al., 1998). A solution of 4 μM Fe (High Purity Standards, SC, United States) and a solution of 4 μM Fe (High Purity Standards) and 80 μM ascorbic acid (Sigma-Aldrich) were used as positive controls to verify the responsiveness of the Caco-2 cells to Fe uptake.

### Statistical Analysis

Statistically significant differences between the NS and 35S-*OsGGP* plants were detected with the two-sample t-test using the 5% level of significance and calculated with Minitab 17.1.0.^1^ The significance of the correlations between ascorbate, phytate, and Fe concentrations in NS and 35S-*OsGGP* BR and GBR with ferritin concentrations in Caco-2 cells were examined with the Pearson correlation method at the 5% significance level using Minitab 17.1.0. A mixed model analysis was performed on the data for each trait from the imaged plants using the R packages ASReml-R (Butler et al., 2018) and asremlPlus (Brien, 2019a). Estimated means were obtained from the analysis, and the significances of their differences were investigated using the Least Significant Difference at the 5% significance level.

## Results

### Constitutive Overexpression of the *OsGGP* Coding Sequence in Rice Increases Ascorbate Concentrations in GBR and in the Vegetative Growth Phase, but Reduces Levels of Ascorbate at the Reproductive Growth Phase

**Figure 1:**
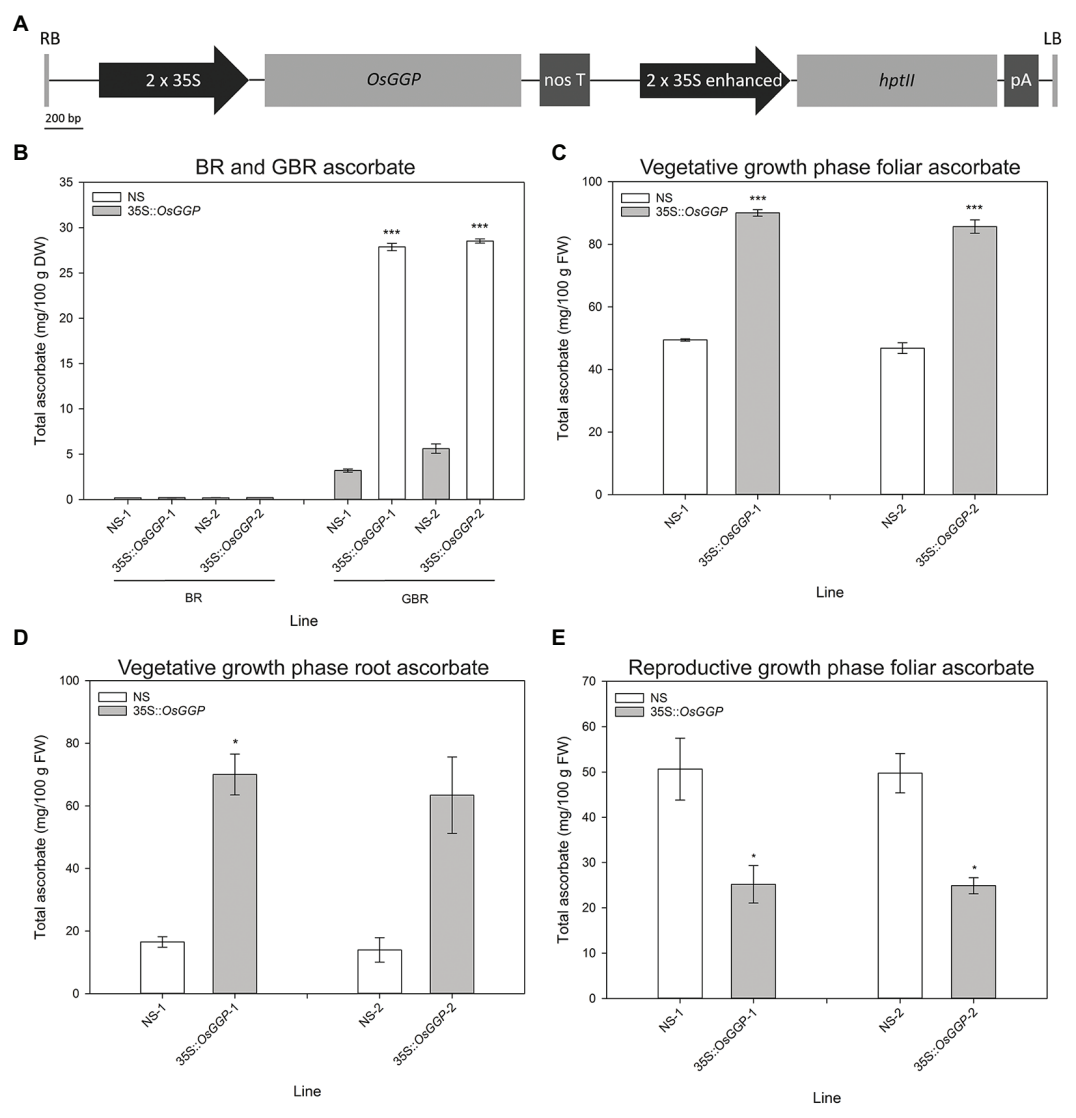
Production and characterization of independent rice transformation events constitutively overexpressing the *OsGGP* coding sequence. **(A)** Schematic representation of the transfer DNA (T-DNA) used for constitutive overexpression of the *OsGGP* coding sequence. RB, right border; 2 × 35S, constitutive dual CaMV 35S promoter; *OsGGP*, *OsGGP* coding sequence; nos T, nopaline synthase terminator; 2 × 35S enhanced, constitutive dual CaMV 35S promoter enhanced; *hptII*, *hygromycin phosphotransferase II*; pA, CaMV poly(A) signal, and LB, left border. **(B)** Ascorbate concentrations of T_3_ homozygous NS and 35S-*OsGGP* brown rice (BR) and germinated brown rice (GBR). Bars represent mean ± SEM of three independent replicates of approximately 50 grain. **(C)** Foliar and **(D)** root ascorbate concentrations of T_3_ homozygous NS and 35S-*OsGGP* plants at the vegetative growth phase [days after planning (DAP) 30]. **(E)** Foliar ascorbate concentrations of T_2_ homozygous NS and 35S-*OsGGP* plants at the reproductive growth phase (DAP 84). Bars represent mean ± SEM of three biological replicates. Asterisks indicate statistically significant differences between NS and 35S-*OsGGP* plants (two-sample *t*-test; ^*^p ≤ 0.05; ^***^p ≤ 0.001).

**Figure 2:**
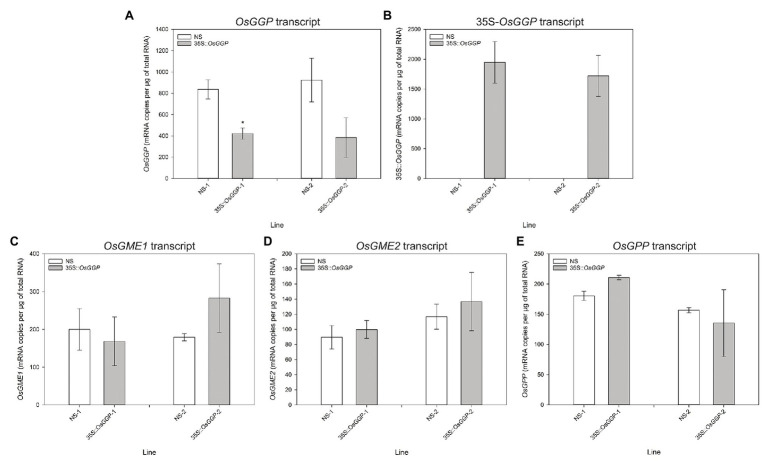
Transcript analysis of null segregant (NS) and 35S-*OsGGP* plants at the reproductive growth phase. Transcript levels of the **(A)** endogenous *OsGGP* gene, **(B)** 35S-*OsGGP* transgene, **(C)** *OsGME1* gene, **(D)** *OsGME2* gene, and **(E)** *OsGPP* gene of T_2_ homozygous NS and 35S-*OsGGP* plants at the reproductive growth phase (DAP 84). Bars represent mean ± SEM of three biological replicates. Asterisks indicate statistically significant differences between NS and 35S-*OsGGP* plants (two-sample *t*-test; ^*^p ≤ 0.05).

Rice cv. Nipponbare transformants constitutively overexpressing the *OsGGP* coding sequence were generated through *Agrobacterium tumefaciens*-mediated transformation of a T-DNA containing the *OsGGP* coding sequence under transcriptional control of the constitutive dual CaMV 35S promoter (Figure 1A). A total of 27 independent, hemizygous 35S-*OsGGP* transformation events were regenerated from tissue culture and ascorbate concentrations were measured in the T_1_ BR. Ascorbate concentrations were negligible in both wild-type (WT) and 35S-*OsGGP* BR but significantly increased up to 5.0-fold in 35S-*OsGGP* GBR relative to WT (Supplementary Table S2). Two events (hereafter referred to as 35S-*OsGGP*-1 and 35S-*OsGGP*-2) with increased ascorbate concentrations in T_1_ GBR and Mendelian segregation ratios of 3:1 for presence/absence of the T-DNA in T_1_ seedling were advanced by selfing and homozygous and NS progeny were identified (Supplementary Table S2). Ascorbate concentrations were negligible in homozygous NS and 35S-*OsGGP* BR and did not differ significantly (Figure 1B). Ascorbate concentrations were, however, significantly increased 8.7-fold and 5.1-fold in homozygous 35S-*OsGGP*-1 and 35S-*OsGGP*-2 GBR relative to NS-1 and NS-2, respectively (Figure 1B). At the vegetative growth phase (defined as germination to panicle initiation Moldenhauer et al., 2001), foliar ascorbate concentrations were significantly increased 1.8-fold in both homozygous 35S-*OsGGP*-1 and 35S-*OsGGP*-2 plants relative to NS-1 and NS-2, respectively (Figure 1C). Similarly, root ascorbate concentrations were significantly increased 4.2-fold and 4.5-fold in homozygous 35S-*OsGGP*-1 and 35S-*OsGGP*-2 plants relative to NS-1 and NS-2, respectively (Figure 1D). In contrast to the vegetative growth phase, foliar ascorbate concentrations at the reproductive growth phase (defined as panicle initiation to heading Moldenhauer et al., 2001) were significantly reduced 2.0-fold in both homozygous 35S-*OsGGP*-1 and 35S-*OsGGP*-2 plants relative to NS-1 and NS-2, respectively (Figure 1E; Supplementary Figure S1). The reduction in foliar ascorbate concentrations in 35S-*OsGGP* plants relative to NS at the reproductive growth phase was only observed in homozygous 35S-*OsGGP* plants; hemizygous 35S-*OsGGP* plants had significantly increased foliar ascorbate concentrations relative to NS at the reproductive growth phase (Supplementary Figure S2). Transcript levels of the endogenous *OsGGP* gene were significantly reduced 2.0-fold in homozygous 35S-*OsGGP*-1 plants relative to NS-1 at the reproductive growth phase (Figure 2A). Similarly, transcript levels of the endogenous *OsGGP* gene were reduced 2.4-fold in homozygous 35S-*OsGGP*-2 plants relative to NS-2 but did not differ significantly (Figure 2A). Even though foliar ascorbate concentrations and endogenous *OsGGP* transcript levels were significantly reduced, high transcript levels of the 35S-*OsGGP* transgene were detected in the 35S-*OsGGP* plants (Figure 2B). Transcript levels of the *GDP-D-mannose-3',5'-epimerase 1* and *2* (*OsGME1* and *OsGME2*) genes, which encode the enzyme responsible for the fourth enzymatic step of the L-galactose pathway, and the *L-galactose-1-phosphate phosphatase* (*OsGPP*) gene, which encodes the sixth enzymatic step of the L-galactose pathway, did not differ significantly between the NS and 35S-*OsGGP* plants (Figures 2C–E).

### Constitutive Overexpression of the *OsGGP* Coding Sequence in Rice Did Not Affect Salt Tolerance at the Vegetative Growth Phase

**Figure 3:**
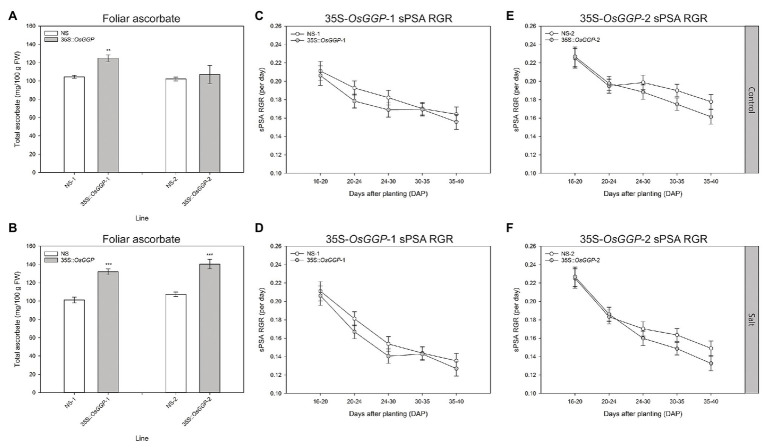
Salt tolerance assessment of NS and 35S-*OsGGP* plants during the vegetative growth phase. Foliar ascorbate concentrations of **(A)** control and **(B)** salt-stressed T_4_ homozygous NS-1 and 35S-*OsGGP*-1 plants and T_3_ homozygous NS-2 and 35S-*OsGGP*-2 plants at DAP 40. Bars represent mean ± SEM of six biological replicates. Asterisks indicate statistically significant differences between NS and 35S-*OsGGP* plants (two-sample *t*-test; ^**^p ≤ 0.01; ^***^p ≤ 0.001). The smoothed projected shoot area (sPSA) relative growth rate (RGR) of **(C)** control and **(D)** salt-stressed T_4_ homozygous NS-1 and 35S-*OsGGP*-1 plants and of **(E)** control and **(F)** salt-stressed T_3_ homozygous NS-2 and 35S-*OsGGP*-2 plants. Salt was applied at DAP 20 and 23. Values represent mean ± half least significant (5%) pairwise difference of six biological replicates. Non-overlapping error bars indicate significant differences at *α* = 0.05.

Shoot growth measurements of control and salt-stressed 35S-*OsGGP* plants during the vegetative growth phase were carried out using automated imaging. Salt stress was applied in two steps to the plants at DAP 20 and DAP 23, and the plants were imaged daily from DAP 14 to DAP 40 inclusive. Under control conditions, foliar ascorbate concentrations were significantly increased 1.2-fold in 35S-*OsGGP*-1 plants relative to NS-1 at DAP 40, however, foliar ascorbate concentrations did not differ significantly between NS-2 and 35S-*OsGGP*-2 plants (Figure 3A). Under salt conditions, foliar ascorbate concentrations were significantly increased 1.3-fold in both 35S-*OsGGP*-1 and 35S-*OsGGP*-2 plants relative to NS-1 and NS-2, respectively, at DAP 40 (Figure 3B). From smoothed PSA (sPSA) values – a strong predictor of shoot biomass – the sPSA absolute growth rate and sPSA relative growth rate (RGA) were calculated at the following DAP intervals: 16–20, 20–24, 24–30, 30–35, and 35–40 (Figures 3C–F; Supplementary Figure S3). As expected, the sPSA RGR declined more rapidly in salt-stressed plants than control plants (Figures 3C–F). However, a similar trend in the sPSA RGR was observed for both control and salt-stressed 35S-*OsGGP*-1 plants relative to NS-1, with the 35S-*OsGGP*-1 plants not differing significantly relative to NS-1 at any of the calculated DAP intervals (Figures 3C,D). Likewise, a similar trend in the sPSA RGR was observed for both control and salt-stressed 35S-*OsGGP*-2 plants relative to NS-2, with the 35S-*OsGGP*-2 plants having a significantly lower sPSA RGR at the DAP interval 30–35 relative to NS-2, but otherwise did not differ significantly for any of the other calculated DAP intervals (Figures 3E,F).

### Ascorbate Concentrations Were Positively Correlated With Ferritin Concentrations in Caco-2 Cells Exposed to *in vitro* Digests of NS and 35S-*OsGGP* BR and GBR Samples

**Figure 4:**
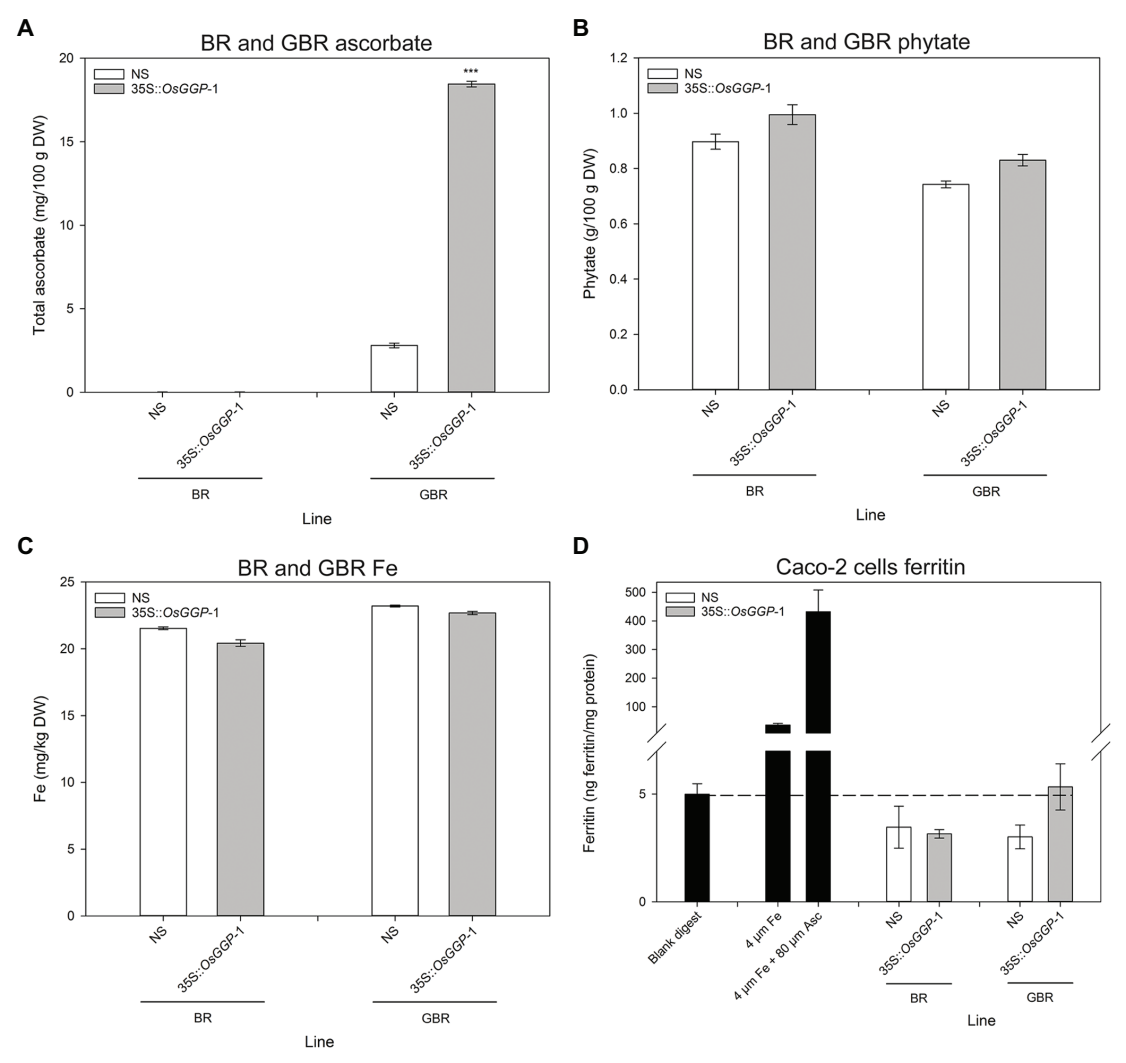
Iron (Fe) bioavailability assessment of NS and 35S-*OsGGP* BR and GBR. Concentrations of **(A)** ascorbate, **(B)** phytate, and **(C)** Fe in T_3_ homozygous NS-1 and 35S-*OsGGP*-1 BR and GBR. Bars represent mean ± SEM of three replicates from a bulked pool of lyophilized grain. **(D)** Ferritin concentrations of Caco-2 cells exposed to *in vitro digests* of T_3_ homozygous NS-1 and 35S-*OsGGP*-1 BR and GBR. A solution of 4 μM Fe and a solution of 4 μM Fe + 80 μM ascorbate (Asc) were used as positive controls to verify the responsiveness of the Caco-2 cells to Fe uptake. Bars represent ± SEM of three biological replicates. Asterisks indicate significant differences between the NS-1 and 35S-*OsGGP*-1 plants (two-sample t-test, ^***^p ≤ 0.001).

The bioavailability of Fe in NS-1 and 35S-*OsGGP*-1 BR and GBR (Supplementary Figure S4) was determined using the coupled *in vitro* digestion/Caco-2 cell culture assay. Ascorbate concentrations were negligible in NS-1 and 35S-*OsGGP*-1 BR and did not differ significantly but were significantly increased 6.6-fold in 35S-*OsGGP*-1 GBR relative to NS-1 (Figure 4A). The concentrations of ascorbate in the NS-1 and 35S-*OsGGP*-1 BR and GBR samples were strongly associated with transcript levels of the endogenous *OsGGP* gene and 35S-*OsGGP* transgene (Supplementary Figure S5). Phytate and Fe concentrations did not differ significantly between NS-1 and 35S-*OsGGP*-1 BR or GBR (Figures 4B,C). The NS-1 and 35S-*OsGGP*-1 BR had molar ratios of 1:31.5:0.0 and 1:38.0:0.0, respectively, of Fe to phytate to ascorbate. The NS-1 and 35S-*OsGGP*-1 GBR had molar ratios of 1:25.3:0.4 and 1:30.3:2.5, respectively, of Fe to phytate to ascorbate. Ferritin concentrations of Caco-2 cells exposed to *in vitro digests* of NS-1 and 35S-*OsGGP*-1 BR were similar and did not differ significantly (Figure 4D). Ferritin concentrations of Caco-2 cells exposed to *in vitro digests* of 35S-*OsGGP*-1 GBR were increased 1.8-fold relative to NS-1 but also did not differ significantly (Figure 4D). Ascorbate concentrations were positively correlated with ferritin concentrations in Caco-2 cells (*r* = 0.620), whereas Fe and phytate concentrations were not (*r* = 0.210 and *r* = 0.042, respectively; Table 1).

**Table 1:** Correlation analysis of ascorbate, phytate, and Fe concentrations in T_3_ homozygous NS-1 and 35S-*OsGGP*-1 BR and GBR with ferritin concentrations in Caco-2 cells exposed to *in vitro* digests of NS-1 and 35S-*OsGGP*-1 BR and GBR samples.

	Fe (μg/g)	Phytate (g/100 g)	Ferritin (ng/mg protein)
Ascorbate (mg/100 g)	0.494	−0.320	0.620^*^
Fe (μg/g)		−0.935^***^	0.210
Phytate (g/100 g)			0.042

Values represent correlation coefficients of 12 replicates. Asterisks indicate significant correlations (Pearson correlation method);

* *p* ≤ 0.05;

*** *p* ≤ 0.001.

## Discussion

Constitutive overexpression of the *OsGGP* coding sequence did not affect ascorbate concentrations in BR, a result that is consistent with the reported decline of ascorbate to negligible levels during grain maturation in cereals (De Gara et al., 2003; Every et al., 2003; Paradiso et al., 2012). In cereal and seed crops, ascorbate concentrations are high up until the start of the dehydration process, after which the levels progressively decline alongside the water content and the ascorbate pool shifts toward the oxidized form; dehydroascorbate (Arrigoni et al., 1992; De Gara et al., 2003; Every et al., 2003; Paradiso et al., 2012). This could reflect an important role for ascorbate in the physiological process of grain dehydration and germination. For example, high doses of ascorbate have been shown to inhibit rice grain germination (Ye et al., 2012), and therefore unless removed from the grain may inhibit germination. Overcoming the decline in ascorbate concentrations during grain maturation represents a significant challenge to ascorbate biofortification of cereal species, such as rice. We therefore suggest that future research in the field of ascorbate biofortification may benefit from focusing on crops that contain meaningful concentrations of ascorbate at the time of harvest, such as fresh fruits and vegetables. Constitutive overexpression of the *OsGGP* coding sequence did, however, significantly increase ascorbate concentrations in GBR (Zhang et al., 2015). To our knowledge, the 8.7-fold increase in ascorbate concentrations in 35S-*OsGGP*-1 GBR, relative to NS-1, represents the largest fold increase in ascorbate reported in any plant tissue to date. It is possible that GBR, an increasingly popular functional food (Patil and Khan, 2011; Cho and Lim, 2016), could serve as an ascorbate biofortified cereal product, but further studies are required to determine the effect(s) of germination conditions, processing, and cooking on the final concentrations of ascorbate, as well as consumer acceptance traits, such as taste and texture. Constitutive overexpression of the *OsGGP* coding sequence also significantly increased ascorbate concentrations in leaves and roots at the vegetative growth phase. The 1.8-fold change in foliar ascorbate concentrations is similar to that reported in previous studies with increased expression of the *Arabidopsis AtGGP* and kiwifruit (*Actinidia chinensis* L.) *AcGGP* gene in rice (Zhang et al., 2015; Ali et al., 2019). To our knowledge, the 4.5-fold increase in ascorbate concentrations in the 35S-*OsGGP*-1 roots, relative to NS-1, represents the largest fold increase reported in plant roots to date and indicates that GGP is likely also a significant rate-limiting enzymatic step toward ascorbate biosynthesis in roots. Contrary to the results in GBR and tissues at the vegetative growth phase, constitutive overexpression of the *OsGGP* coding sequence significantly reduced foliar ascorbate concentrations at the reproductive growth phase. This reduction in ascorbate concentrations was dependent on homozygosity of the 35S-*OsGGP* transgene and was associated with reduced transcript levels of the endogenous *OsGGP* gene. This data suggest that post-transcriptional silencing of the endogenous *OsGGP* gene and 35S-*OsGGP* transgene may be occurring in response to elevated transcript levels of *OsGGP* in a homozygous-dependent manner (James et al., 2002). Detection of high transcript levels of the 35S-*OsGGP* transgene does not necessarily fit the traditional model of post-transcriptional gene silencing, however, similar observations were made when the kiwifruit *AcGGP* gene was expressed in *Arabidopsis*, and was interpreted as incomplete processing of the silenced gene transcripts, thereby leaving mRNA template for detection by qRT-PCR (Bulley et al., 2009). The identification of *OsGGP* derived small interfering RNAs, for example, may indicate the occurrence of gene silencing in the 35S-*OsGGP* plants and warrants further investigation. Future efforts to increase ascorbate concentrations in plants could avoid the use of transgene overexpression by utilizing genome editing tools such as the CRISPR/Cas9 system to disrupt transcriptional or translational repressors of ascorbate biosynthetic genes, such as the highly conserved *cis*-acting upstream open reading frame that controls translation of the *GGP* gene (Laing et al., 2015; Bulley and Laing, 2016; Macknight et al., 2017; Li et al., 2018; Zhang et al., 2018; Broad et al., 2019, 2020; Si et al., 2020).

We also examined whether the 35S-*OsGGP* plants displayed altered salt tolerance at the vegetative growth phase using automated imaging. Previous studies have demonstrated that non-destructive measurement such as sPSA is a strong predictor of shoot biomass, and that the sPSA RGR can be used effectively to screen for salt tolerance in rice (Hairmansis et al., 2014; Campbell et al., 2015; Al-Tamimi et al., 2016; Yichie et al., 2018). However, a similar trend in the sPSA, RGR was observed for both control and salt-stressed 35S-*OsGGP* plants relative to NS at all calculated DAP intervals, suggesting that the elevated ascorbate concentrations from constitutive overexpression of the *OsGGP* coding sequence in rice did not affect salt tolerance in terms of shoot biomass during this early growth phase. This result is incongruent with many previous studies that have reported enhanced salt tolerance in model and crop species with increased expression of ascorbate biosynthetic genes (Hemavathi et al., 2009, 2010, 2011; Upadhyaya et al., 2011; Zhang et al., 2011, 2015; Lim et al., 2012, 2016b; Lisko et al., 2013; Liu et al., 2013; Ma et al., 2014; Cai et al., 2015; Ali et al., 2019). Particularly, our results differ with a previous study that found that increased expression of the *Arabidopsis AtGGP* gene in rice to enhance seedling salt tolerance in terms of biomass and RGRs (Zhang et al., 2015). Our results may be more consistent with a recent study that reported that increased expression of the kiwifruit *AcGGP* gene in rice enhanced salt tolerance in terms of visible symptoms and lipid peroxidation, but not shoot biomass (Ali et al., 2019). Investigating oxidative stress markers such as lipid peroxidation in the salt-stressed 35S-*OsGGP* plants may determine whether constitutive overexpression of the *OsGGP* coding sequence in rice ameliorates salt-induced oxidative stress and will be the subject of future research. Limitations of this study include testing only one salt concentration and a relatively low fold increase in foliar ascorbate concentrations in the 35S-*OsGGP* plants at DAP 40, therefore making it difficult to draw definitive conclusions regarding the contribution of increased ascorbate on salt tolerance in rice. It is worth noting, however, that ascorbate concentrations may have been significantly higher during earlier intervals of the experiment, for example when the salt application was applied at DAP 20 and 23, as we had previously detected a 1.8- and 4.5-fold increase in foliar and root ascorbate concentrations, respectively, in the 35S-*OsGGP* plants measured at DAP 30; presented in Figures 1C,D. As we did not detect any significant differences in the sPSA RGR for any of the calculated DAP intervals following salt application, our data suggest that ascorbate does not affect salt tolerance in terms of shoot biomass during this early growth phase of rice. Future research will focus on the production of rice with increased ascorbate concentrations at all stages of development, as well as testing a range of salt concentrations to identify any effects that increased ascorbate concentrations may have on salt tolerance in rice.

The ascorbate-enriched 35S-*OsGGP* GBR that we generated in this study presented an opportunity to test, for the first time, whether engineering ascorbate-enriched crops could improve Fe bioavailability in human digestion utilizing the Caco-2 cell line model – an accurate predictor of human Fe absorption (Glahn et al., 1998). Previous studies have reported that phytate significantly reduces Fe bioavailability, beginning at a 1:1 molar ratio of Fe to phytate, with maximal inhibition occurring at a ratio around 1:10 (Glahn et al., 2002; Engle-Stone et al., 2005; Jin et al., 2008). Ferritin concentrations below or equivalent to the blank digest for the Caco-2 cells exposed to *in vitro* digests of NS-1 and 35S-*OsGGP*-1 BR and GBR in this study can therefore likely be explained by the high levels of phytate (Glahn et al., 2002; Jin et al., 2008). We found that ferritin concentrations of Caco-2 cells exposed to *in vitro* digests of the ascorbate-enriched 35S-*OsGGP*-1 GBR were increased 1.8-fold relative to NS-1 but did not differ significantly, despite a significant improvement in the molar ratio of Fe to phytate to ascorbate. This experiment highlights the difficulty of increasing the bioavailability of Fe from unpolished rice grain and suggests that even greater increases in ascorbate levels or greater reductions in phytate levels are required to significantly improve the bioavailability of the Fe. One limitation of our study is that we did not investigate the concentrations of phenolic compounds – that are known to inhibit Fe absorption – in the BR and GBR (Glahn et al., 2002; Engle-Stone et al., 2005; Jin et al., 2008). Total phenolic compounds have been reported to increase in GBR, and thus it is possible that increased phenolic compounds may have reduced the promoting effects of ascorbate on Fe bioavailability in the 35S-*OsGGP* GBR (Wei et al., 2013). We did, however, find that ascorbate concentrations – but not Fe or phytate concentrations – positively correlated with Caco-2 cell ferritin formation in our study. This is consistent with an *in vitro* bioavailability study of Andean potato clones with varying Fe, ascorbate, and phenolic concentrations that reported that ascorbate concentrations were the strongest predictor of ferritin formation (Andre et al., 2015). As our study is inconclusive with respect to determining whether increasing ascorbate concentrations in crops could help improve Fe bioavailability in human digestion, further *in vitro* and *in vivo* assessments of ascorbate-enriched crops on Fe bioavailability are necessary and should be the focus of future research efforts. While many cereal crops, such as rice, may not be suitable candidates for increasing Fe bioavailability through enhanced ascorbate concentrations due to the observed decline in ascorbate levels in maturing grain (De Gara et al., 2003; Every et al., 2003; Paradiso et al., 2012), many other staple crops may be suitable. For example, sweet potato (*Ipomoea batatas* L.) and cassava (*Manihot esculenta*), the two vegetable crops targeted for Fe biofortification so far (Bouis et al., 2011), both possess meaningful ascorbate levels at harvest and could be excellent candidates for ascorbate enrichment alongside Fe biofortification strategies.

## Data Availability Statement

The raw data supporting the conclusions of this article will be made available by the authors, without undue reservation.

## Author Contributions

JB, RH, and AJ conceived, designed and supervised the research. RB, JB, JB, SR, and PS performed the research. RB, NJ, and CB curated and analyzed the data. BB, ET, RG, and RH contributed resources and project administration. RB and RG acquired funding. RB drafted the manuscript. All authors contributed to the article and approved the submitted version.

### Conflict of Interest

The authors declare that the research was conducted in the absence of any commercial or financial relationships that could be construed as a potential conflict of interest.

## Footnotes

^1^http://www.minitab.com/en-us/
