# Supplementary material for: Effect of Rice GDP-L-Galactose Phosphorylase Constitutive Overexpression on Ascorbate Concentration, Stress Tolerance, and Iron Bioavailability in Rice
Source: Front Plant Sci. 2020 Dec 3;11:595439. doi: 10.3389/fpls.2020.595439 (PMC7744345; doi:10.3389/fpls.2020.595439)
Supplement: Supplementary file 1 [file Table_1.docx]

Supplementary Material

| **SUPPLEMENTARY TABLE 1 \|** List of PCR and qRT-PCR primers used in this study. The table provides primer name, forward and reverse primer sequences, PCR product length, and the application of the primers. | | | | |
| --- | --- | --- | --- | --- |
| **Primer name** | **Forward primer sequence (5' - 3')** | **Reverse primer sequence (5' - 3')** | **Product length (bp)** | **Application** |
| *OsGGP* | ATGGAGATGAAGCTGACCATCAAG | CTGCCTCGTCCTTCAGTGA | 1317 | Cloning the *OsGGP* coding sequence |
| *OsACT1* | TCAGCAACTGGGATGATATGGAG | GCCGTTGTGGTGAATGAGTAAC | 634 | Detecting the 35S-*OsGGP* T-DNA |
| *hptII* | CTTGTATGGAGCAGCAGACGC | CTATTTCTTTGCCCTCGGACG | 308 | Detecting the 35S-*OsGGP* T-DNA |
| qRT-PCR-35S-*OsGGP* | AGGAAGTCAGCGAAGACGAG | AAGACCGGCAACAGGATTC | 249 | Transcript analysis of the 35S-*OsGGP* transgene |
| qRT-PCR-*OsGGP* | AGATCTTCCTCTTCCCTCAGT | CCGACAACCTGTTCTACCGT | 368 | Transcript analysis of the endogenous *OsGGP* gene |
| qRT-PCR-*OsGME1* | CGTATCAATGGTGTGAAGAGGTT | GGCCAGGCATCAGATTCCTT | 107 | Transcript analysis of the *OsGME1* gene |
| qRT-PCR-*OsGME2* | GATATGGGAGGGATGGGATT | GCCAAGCATCTGACTCCTTC | 196 | Transcript analysis of the *OsGME2* gene |
| qRT-PCR-*OsGPP* | TGCCTTCCTCAATGGTTCTC | CACACATGTTCAAGGCCAAC | 188 | Transcript analysis of the *OsGPP* gene |
| qRT-PCR-*OsGAPDH* | GGGCTGCTAGCTTCAACATC | TTGATTGCAGCCTTGATCTG | 190 | qRT-PCR housekeeping gene |
| qRT-PCR-*OsELF1* | GAAAGCAGCAGAAGAACGGG | TCCTCAAGCTTTGCCATGTCT | 126 | qRT-PCR housekeeping gene |
| qRT-PCR-*OsACT1* | ATCACTGCCTTGGCTCCTAG | TCGTACTCAGCCTTGGCAA | 140 | qRT-PCR housekeeping gene |
| qRT-PCR-*OsUBQ5* | TACACCAAGCCCAAGAAGCAG | GTGAGGCCGCACTTACCG | 191 | qRT-PCR housekeeping gene |

| **SUPPLEMENTARY TABLE 2 \|** Ascorbate concentrations of WT and T_1_ 35S-*OsGGP* germinated brown rice and the number of T_1_ 35S-*OsGGP* seedlings with the 35S-*OsGGP* T-DNA present or absent. | | | | |
| --- | --- | --- | --- | --- |
| **Genotype** | **Total ascorbate (mg/100 g DW)^a^** | **No. of seedlings with T-DNA** | **No. of seedlings without T-DNA** | **Chi-square p-value^b^** |
| WT | 1.66 | - | - | - |
| Event 1 | 4.35 | 8 | 2 | 0.715 |
| Event 2 | 2.88 | 10 | 2 | 0.505 |
| Event 4 | 2.37 | 10 | 1 | 0.223 |
| Event 5 | 2.94 | 11 | 1 | 0.182 |
| Event 6 | 3.07 | 9 | 2 | 0.602 |
| Event 7 | 5.64 | 10 | 2 | 0.505 |
| Event 8 | 4.44 | 8 | 4 | 0.505 |
| Event 10 | 3.82 | 11 | 1 | 0.182 |
| Event 13 | 4.10 | 11 | 1 | 0.182 |
| Event 16 | 2.31 | 11 | 1 | 0.182 |
| Event 19 | 2.62 | 11 | 1 | 0.182 |
| Event 20 (35S-*OsGGP*-2) | 5.19 | 8 | 2 | 0.715 |
| Event 22 | 2.17 | 9 | 1 | 0.273 |
| Event 24 | 5.36 | 9 | 1 | 0.273 |
| Event 25 | 6.29 | 10 | 1 | 0.223 |
| Event 26 | 5.18 | 8 | 2 | 0.715 |
| Event 27 | 6.26 | 10 | 1 | 0.223 |
| Event 30 | 1.84 | 10 | 1 | 0.223 |
| Event 33 | 0.95 | 8 | 0 | 0.103 |
| Event 41 | 2.26 | 11 | 1 | 0.182 |
| Event 42 | 4.07 | 7 | 1 | 0.414 |
| Event 43 | 3.15 | 8 | 3 | 0.862 |
| Event 45 | 2.52 | 10 | 1 | 0.223 |
| Event 46 | 6.11 | 12 | 0 | 0.046* |
| Event 49 | 4.22 | 10 | 2 | 0.505 |
| Event 50 | 3.74 | 11 | 1 | 0.182 |
| Event 51 (35S-*OsGGP*-1) | 8.30 | 10 | 2 | 0.505 |
| ^a^Total ascorbate values represent one replicate of four to eleven grain. ^b^Asterisks indicate statistically significant differences between observed and expected segregation ratio of 3:1 for the presence to absence of the 35S-*OsGGP* T-DNA (chi-square test; * p-value ≤0.05). | | | | |


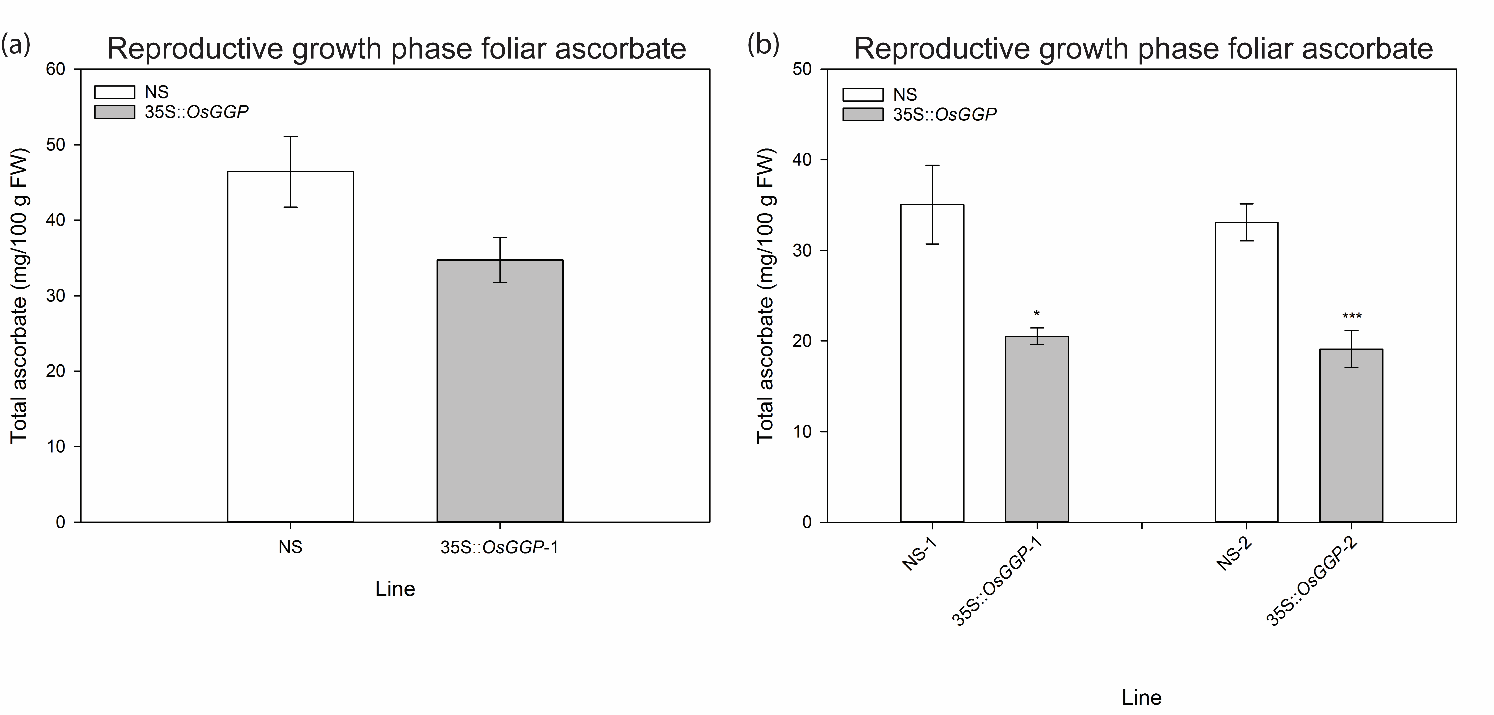


**SUPPLEMENTARY FIGURE 1 |** Foliar ascorbate concentrations of T_3_ and T_4_ NS and 35S-*OsGGP* plants at the reproductive growth phase. **(A)** Foliar ascorbate concentrations in T_3_ homozygous 35S-*OsGGP*-1 plants at the reproductive growth phase (DAP 84). Bars represent mean ± SEM of three biological replicates. (**B**) Foliar ascorbate concentrations of T_4_ homozygous NS-1 and 35S-*OsGGP*-1 and T_3_ NS-2 and 35S-*OsGGP*-2 plants at the reproductive growth phase (DAP 84). Bars represent mean ± SEM of five to six biological replicates. Asterisks indicate statistically significant differences between NS and 35S-*OsGGP* plants (two-sample t-test; * p-value ≤0.05; *** p-value ≤0.001).

**SUPPLEMENTARY FIGURE 2 |** Foliar ascorbate concentrations in T_1_ hemizygous and homozygous NS and 35S-*OsGGP* plants at the reproductive growth phase (DAP 84). Bars represent mean ± SEM of one to seven biological replicates. Asterisks indicate statistically significant differences between NS and 35S-*OsGGP* plants (two-sample t-test; ** p-value ≤0.01; *** p-value ≤0.001).


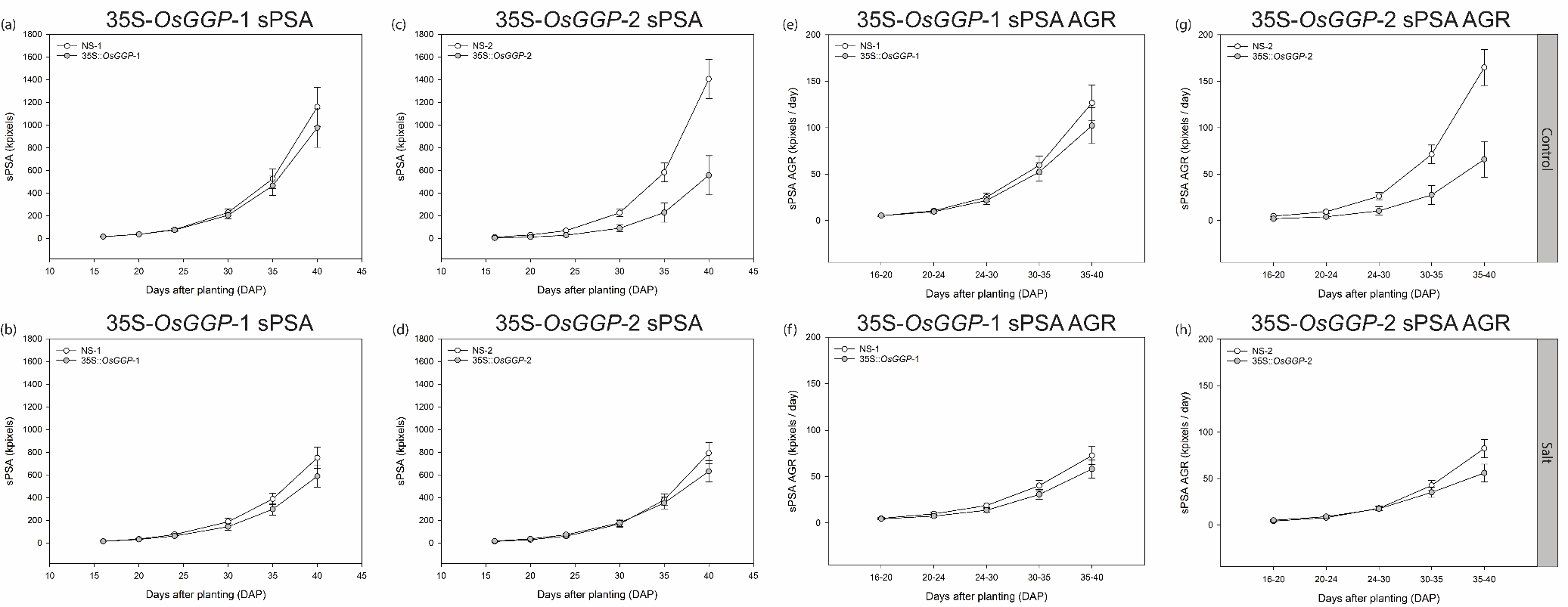


**SUPPLEMENTARY FIGURE 3** **|** The sPSA and sPSA absolute growth rate of control and salt-stressed NS and 35S-*OsGGP* plants during the vegetative growth phase. The sPSA of (**A**) control and (**B**) salt-stressed T_4_ homozygous NS-1 and 35S-*OsGGP*-1 plants and of (**C**) control and (**D**) salt-stressed T_3_ homozygous NS-2 and 35S-*OsGGP*-2 plants. The sPSA absolute growth rate (AGR) of (**E**) control and (**F**) salt-stressed T_4_ homozygous NS-1 and 35S-*OsGGP*-1 plants and of (**G**) control and (**H**) salt-stressed T_3_ homozygous NS-2 and 35S-*OsGGP*-2 plants. Salt was applied at DAP 20 and 23. Values represent mean ± half least significant pairwise (5%) difference of six biological replicates. Non-overlapping error bars indicate significant differences at α = 0.05.

**SUPPLEMENTARY FIGURE 4 |** Images of the germinated brown rice used in this study. (**A**) NS-1 and (**B**) 35S-*OsGGP*-1 germinated brown rice.


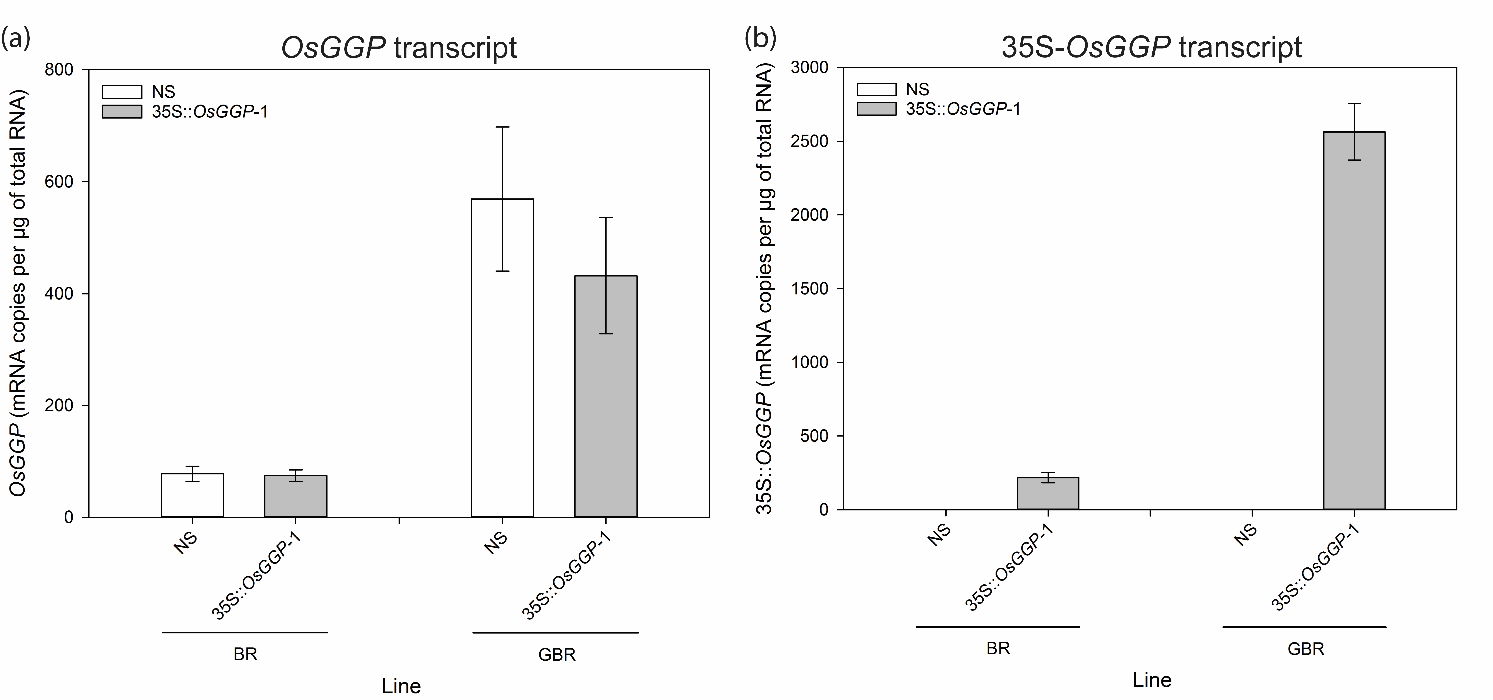


**SUPPLEMENTARY FIGURE 5 |** Transcript analysis of the endogenous *OsGGP* gene and 35S-*OsGGP* transgene in NS and 35S-*OsGGP* brown rice and germinated brown rice. Transcript levels of the (**A**) endogenous *OsGGP* gene and (**B**) 35S-*OsGGP* transgene in T_3_ homozygous NS-1 and 35S-*OsGGP*-1 brown rice (BR) and germinated brown rice (GBR). Bars represent mean ± SEM of three independent replicates of subsampled grain, each with four technical replicates. No statistically significant differences were detected between NS and 35S-*OsGGP* plants (two-sample t-test).
